# A novel optimal formula of nickel extraction: arsenic removal from niccolite by controlling arsenic-containing phases

**DOI:** 10.3389/fchem.2023.1290831

**Published:** 2023-12-07

**Authors:** Xiaowei Tang, Yuehui He

**Affiliations:** ^1^ Powder Metallurgy Research Institute, Central South University, Changsha, China; ^2^ State Key Laboratory of Powder Metallurgy, Central South University, Changsha, China

**Keywords:** nickel, arsenic, niccolite, roasting, S, FeS

## Abstract

**Objective:** Niccolite, a rare nickel arsenide mineral, has emerged as a promising source for nickel extraction. However, its processing is limited and often associated with toxicity concerns. This study aims to search for efficient separation of arsenic during the roasting process of niccolite.

**Methods:** The arsenic-containing phase was optimized through changing the contents of oxygen, additive S, and additive FeS in the system to achieve efficient separation of arsenic during the roasting process of niccolite. Thermodynamic analysis was performed using the equilibrium composition module with HSC Chemistry.

**Results:** The thermodynamic results showed that in direct roasting, the product contained ferric arsenate which immobilized arsenic in the solid phase, increasing the difficulty in separation. In the presence of sulfur, the arsenic may escape completely in the form of gas (As_2_O_3_, As_4_O_4_, As_4_O_6_). The use of FeS as the reductant significantly reduced the residual arsenic content.

**Conclusion:** The FeS reduction in roasting process is an optimal strategy for arsenic removal from niccolite. This provides a novel technique for nickel extraction in industry.

## 1 Introduction

Nickel is an important strategic metal with widespread applications in stainless steel and clean energy fields (Pariser et al., 2018; Poole et al., 2022). The consumption of nickel has reached 2.89 million tons in 2022 and the International Nickel Study Group (INSG) expects this to reach 3.22 million tons in 2023 (INSG, 2013). In recent years, the growth of nickel consumption has mainly come from the increasing demand for power batteries, which has reached 15% of the total consumption of nickel (Wang et al., 2022; Zhou et al., 2022). China alone accounts for close to 52% of world nickel demand. Annual production of electric vehicles (comprising 49%–60% Ni wt%) will reach 31 million in 2025, increasing high pure demand from 33 kt in 2017 to 570 kt in 2025 (Meshram et al., 2019). However, the world reserves of nickel are estimated at 74 million tons of Ni metal content (U.S.-Geological-Survey, 2018). Australia (25%), Brazil (16%), Russia (10%), Cuba (7%), Indonesia (6%), South Africa (5%), Canada (3.6%) and China (3.9%) together account for around 75% of the nickel reserves. The nickel mined comes from two types of ore deposits, magmatic sulfide deposits and laterites. Sulfide nickel ore, due to a long-term excavation, has seen a constant decline in production in China (Murofushi et al., 2022), and more than 60% of nickel ore is laterite nickel ore (Pandey et al., 2023) presently. Nevertheless, due to Indonesia’s embargoes on raw ore exports (Hobson, 2013; Shi and Ju, 2023), diminishing production from Philippines’ mines (Lee, 2017), and the Russian-Ukrainian war causing significant restrictions on the export of nickel (Erickson, 2022), the price of nickel ore shows an overall upward trend, with the discord between supply and demand becoming increasingly conspicuous. Thus, certain minerals, which were formerly deemed to be of low value, such as nickel arsenide ore, have become prospective contenders for nickel extraction.

Niccolite (NiAs), an ore mineral of nickel, is commonly associated with other nickel arsenides and sulfides, and classified in a group of sulfide minerals (Liu et al., 2023). NiAs is rarely used as a source of nickel due to the presence of arsenic, which is deleterious to most smelting and milling techniques. When nickel sulfide ore deposits are altered to produce NiAs, the presence of arsenic often renders the ore uneconomic when concentrations of As reach several hundred parts per million. However, arsenic bearing nickel ore may be treated by blending with “clean” ore sources, to produce a blended feedstock that can be handled with acceptable recovery by the mill and smelter. Thus, as a general rule, smelters do not accept ores containing more than 4% arsenic (China, 2015). Presently, the treatment of NiAs is primarily involved in flotation process (Iwasaki et al., 1986; Nakazawa and Iwasaki, 1986b; a; Dai et al., 2005; Senior et al., 2009). This method causes a significant water pollution, and does not effectively utilize nickel and arsenic.

To address the above problem, a method of arsenic removal from red arsenic nickel ore was proposed using sulfur roasting in the present study. In this methods, pure oxidized arsenic can be extracted by filtering the dust with porous materials. Currently, the application and development of porous intermetallic materials in gas-solid separation has proven to be an effective solution to the toxicity problem of arsenic bearing mineral (Zhang et al., 2017). The thermal stability, corrosion resistance, and high toughness of this material enable it to directly filter the high-temperature flue gas generated by the roasting process (Gao et al., 2009; Sheng et al., 2010; Jiang et al., 2018). Through filtration of dust at high-temperature and condensed arsenic compounds at low-temperature, this material may separate and extract arsenic elements from the source of smelting.

The aim of this study is to explore an arsenic removal from NiAs by controlling arsenic-containing phases. The process of arsenic removal by sulfur roasting was proposed by comparing thermodynamic data, and additions of S and FeS were verified a suggestion methods.

## 2 Raw materials and methods

### 2.1 Raw materials

The raw materials used in the experiment came from the mines in Yunnan. The results of mineral elements detected by ICP showed that the main elements were As (18.6 wt%) and Ni (16.1 wt%). The mineral composition was analyzed by XRD, and the main mineral components were NiAs (34.7 wt%) and SiO_2_ (65.3 wt%).

### 2.2 Thermodynamic approaches

Thermodynamic analysis was performed using the equilibrium composition module in HSC Chemistry^®^ 6.0. The thermodynamic data of specific substances were obtained from database module of HSC and CRC handbook of chemistry and physics (Internet Version 2016) (96th). Specifically, the Gibbs free energy (GFE) of all reactions can be calculated by Formula 1 and the occurrence of the reaction in the corresponding temperature range can be determined by Formula 1.

$$\Delta_{r}G_{m}\left(T\right)=\Delta_{r}H_{m}\;\left(T\right)-\;T\Delta_{r}S_{m}\;\left(T\right)$$ (1)

In the equation, Δ_r_G_m_ is GFE, Δ_r_H _m_ is enthalpy, Δ_r_S _m_ is entropy, and T is temperature.

The relationship between GFE and reactants was established by Formula 2.

$$\Delta_{r}G_{m}\left(T\right)=\Delta_{r}G_{m}^{\theta }\left(T\right)+\text{RTlnQ}$$ (2)

When the reaction is in equilibrium, Δ_r_G_m_(T) = 0 and Q = k^θ^,

thus Formula 3 can be derived.

$$\Delta_{r}G_{m}^{\theta }\left(T\right)=-{\text{RTlnk}}^{\theta }$$ (3)

In the equation, Δ_r_G_m_^θ^ is standard GFE, R is molar gas constant, Q is reaction quotient, and k^θ^ is equilibrium quotient.

k^θ^, as derived from Formula 4, is the ratio of the concentration entropy of the products to that of the reactants at equilibrium, and the gas components are the ratio of partial pressures.

Kθ=ПPj^njproduct/ ПPi ^nireactant (4)

In the equation, P is partial pressures, j is product, i is reactant, and n is chemical coefficient.

Finally, Formula 5 was used to obtain the ratio of molar amounts of the gas components as the ratio of partial pressures:

PV=nRT (5)

By combining the above formulas, the equilibrium molar amount of each component of the reactants at a specific temperature may be calculated step by step.

### 2.3 TG/DTA method

Concurrent TG/DTA (SDT 650, WATERS CORPORATION) was used to identify various reactions on the basis of weight changes as well as thermal effects. Tests were done under flowing nitrogen or air and at a heating rate of 20°C/min.

### 2.4 Experimental method

**FIGURE 1:**
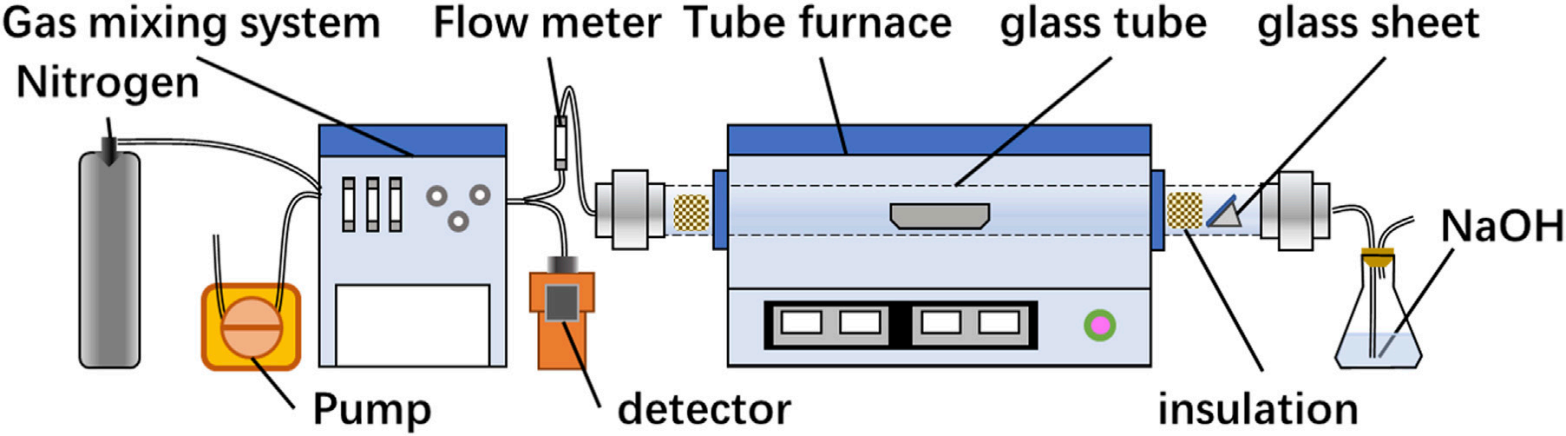
Roasting experimental setup.

A schematic diagram of the roasting setup is presented in Figure 1. The air was pumped into the gas mixing system through a peristaltic pump and combined with nitrogen. The resultant mixture was then divided into two parts, one part entering the gas detector for oxygen content detection and other part entering the tube furnace, regulated by the gas flow meter. For each experiment, the crucible containing a mineral of the requisite mass was placed in a furnace and heated to the specified temperature. The glass pane was situated behind the insulation to collect the volatilized condensate from the concentrate. Subsequently, the gas was purified using a sodium hydroxide solution before being discharged.

## 3 Results and discussion

### 3.1 The limitation of direct roasting

#### 3.1.1 Reaction mechanism

Direct roasting refers to adjusting the reaction only by controlling the oxygen content without addition.

**FIGURE 2:**
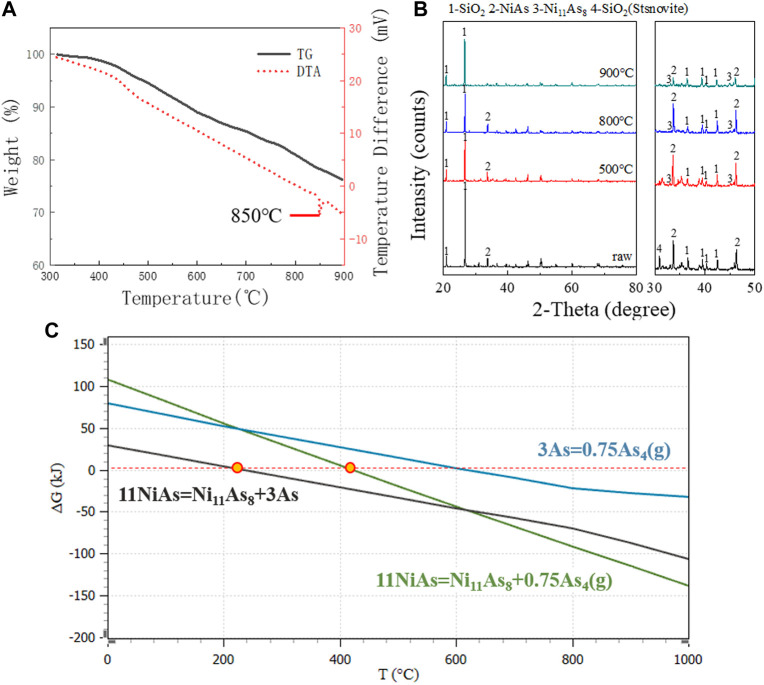
DTA, TG and XRD images in anaerobic conditions. **(A)** TG/DTA diagram for raw ore in nitrogen. **(B)** XRD patterns for raw ore roasting at 900°C, 800°C, and 500°C. **(C)** GFE for decomposition reaction of NiAs.

In anaerobic conditions, DTA and TG images showed a continuous decrease after 300°C (Figure 2A). The XRD images showed that Ni_11_As_8_ was generated at 500°C, 800°C and 900°C (Figure 2B). According to the report of L.J Wilson (Wilson and Mikhail, 1987), Ni_11_As_8_ decomposes according to reaction (6).

$$11\text{NiAs}={\text{Ni}}_{11}{\text{As}}_{8}+3/4{\text{As}}_{4}\left(g\right)$$ (6)

However, by comparing the XRD images, it was found that the peak of NiAs changed little at 500°C and 800°C, and the peak of NiAs decreased significantly when the temperature reached 900°C. This indicates that the main temperature of reaction (6) is above 800°C. The DTA image showed that there is an endothermic peak around 850°C, which corresponds to the boiling temperature of Arsenic (816°C). Finally, by comparing the GFE (Figure 2C) of reaction, the reaction of NiAs decomposing to produce As started at about 248°C, while the reaction of NiAs decomposing to produce As_4_(g) started at about 420°C. Comparing the two reactions, the reaction of NiAs decomposing to produce As was more consistent with the process of weight loss shown by the TG image. And the GFE of NiAs decomposition reaction was less than that of As volatilization reaction. The above evidence shows that NiAs decomposition may be divided into two reactions (7) and (8).

$$11\text{NiAs}={\text{Ni}}_{11}{\text{As}}_{8}+3\text{As}$$ (7)

$$4\text{As}={\text{As}}_{4}\left(g\right)$$ (8)

**FIGURE 3:**
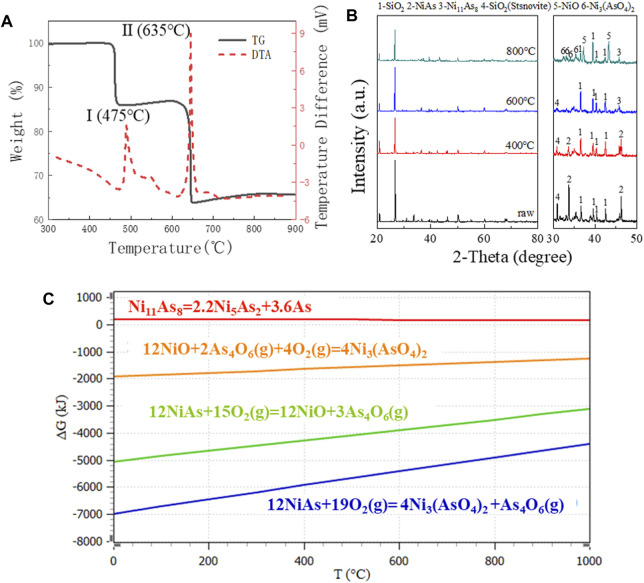
TG diagram, XRD pattern and GFE data in oxygen presence at various temperatures. **(A)** TG/DTA diagram for raw ore in air. **(B)** XRD pattern for raw ore roasting in 800°C, 600°C, and 400°C. **(C)** GFE for oxidation reaction of NiAs.

In the presence of oxygen, the thermal activities which appear on the DTA diagram and the corresponding weight changes on the TG diagram of Figure 3A may be explained as follows.

**Peak I,** The oxidation of NiAs in air started around 475°C, and resulted in a substantial exothermic peak. XRD results (Figure 3B) indicated that the reaction product associated with peak I is Ni_11_As_8_, and the reaction at this stage may be represented by the following equation.

$$11\text{NiAs}+9/4O_{2}\left(g\right)={\text{Ni}}_{11}{\text{As}}_{8}+3/4{\text{As}}_{4}O_{6}\left(g\right)$$ (9)

It is possible that the reaction (9) occurs via a two-step mechanism which consists of an endothermic thermal dissociation of NiAs reaction (7) and a highly exothermic reaction between As_4_ and O_2_(g) to form As_4_O_6_(g) vapour. However, the reaction free energy profile suggests that the GFE of the reaction between As_4_ and O_2_(g) to form As_4_O_6_(g) is considerably lower than that of reaction (7), indicating that As_4_ would be oxidized immediately upon formation, thus reaction (9) should be regarded as a whole.

**Peak Ⅱ,** When the temperature exceeded 635°C, the second peak of DTA was generated, while the TG experienced a significant weight loss. XRD results indicated that the reaction product associated with peak I was NiO and Ni_3_(AsO_4_)_2_ (Figure 3B), at this stage, it may be represented by the following Eqs 10, 11.

$${\text{Ni}}_{11}{\text{As}}_{8}+23/2O_{2}\left(g\right)=11\text{NiO}+2{\text{As}}_{4}O_{6}\left(g\right)$$ (10)

$$6\text{NiO}+{\text{As}}_{4}O_{6}\left(g\right)+2O_{2}\left(g\right)=2{\text{Ni}}_{3}{\left({\text{AsO}}_{4}\right)}_{2}$$ (11)

The reaction (10) occurs through a two-step mechanism which consists of an endothermic thermal dissociation of Ni_11_As_8_ and oxidation of Ni_5_As_2_. However, the reaction GFE data suggested that the GFE of dissociation of Ni_11_As_8_ to Ni_5_As_2_ was higher than zero, indicating that the reaction did not proceed spontaneously and the presence of Ni_5_As_2_ cannot be found in the XRD image, thus the reaction cannot be regarded as two steps. Meanwhile, due to the low GFE of formation of Ni_3_(AsO_4_)_2_, Ni_3_(AsO_4_)_2_ may definitely be generated during NiAs oxidation (Figure 3C).

**FIGURE 4:**
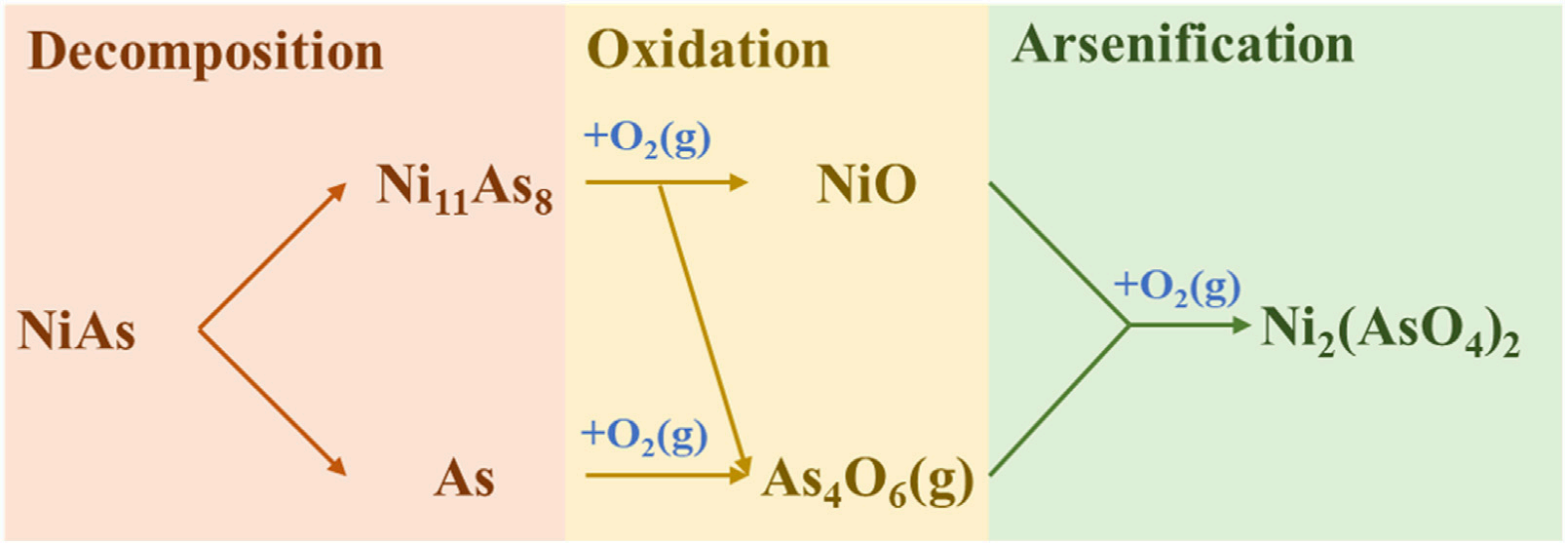
Reaction mechanism of direct roasting.

In summary, the direct oxidation process of NiAs may be concluded into three stages (Figure 4). Firstly, the decomposition, where part of the As in NiAs volatilizes to form Ni_11_As_8_. Secondly, oxidation, where the oxidation of As yields As_4_O_6_ and the oxidation of Ni_11_As_8_ yields NiO and As_4_O_6_. Lastly, the excessive oxidation of NiO yields Ni_3_(AsO_4_)_2_.

#### 3.1.2 Thermodynamic equilibrium component

**FIGURE 5:**
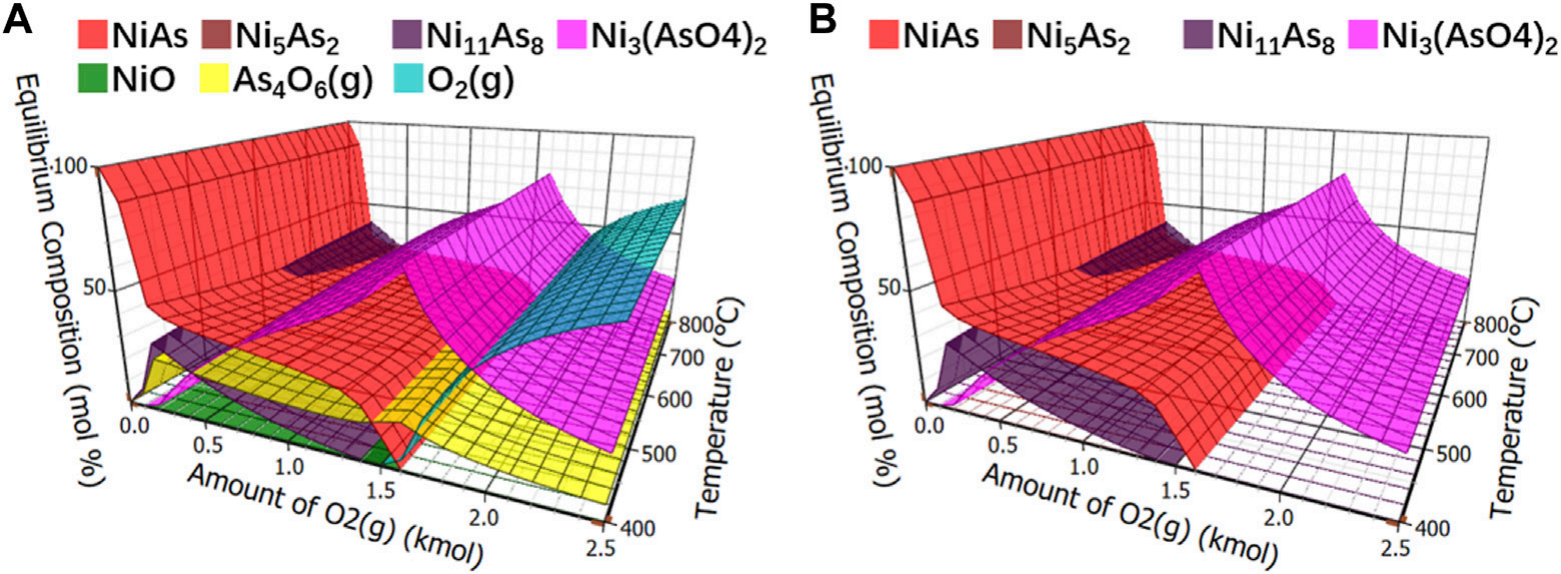
Calculation of equilibrium components in oxidation roasting. **(A)**, Equilibrium component of oxidative roasting. **(B)**, Equilibrium component of arsenide nickel and arsenate nickel in oxidative roasting.

Controlling the amount of oxygen is the most direct method for controlling oxygen content. The initial quantity of thermodynamic equilibrium components was 1 kmol NiAs, and different contents of oxygen were added to observe the content of products in mol percentage at different temperatures. The calculation results of components in oxidation roasting balance were shown in Figure 5A, which are relatively complex. In order to facilitate observation, other compounds were removed, and arsenide nickel and arsenate nickel were simplified, as shown in Figure 5B.

In Figure 5B, arsenide nickel and arsenate nickel were overlapped. Under low oxygen condition, the reaction of NiAs was not complete, and the product Ni_11_As_8_ still contained a large amount of arsenic. When the oxygen level was slightly increased, it may generate the difficult-to-volatilize Ni_3_(AsO_4_)_2_. Therefore, arsenate nickel or arsenide nickel is definitely produced in the oxidation roasting process.

#### 3.1.3 Roasting experiment

**FIGURE 6:**
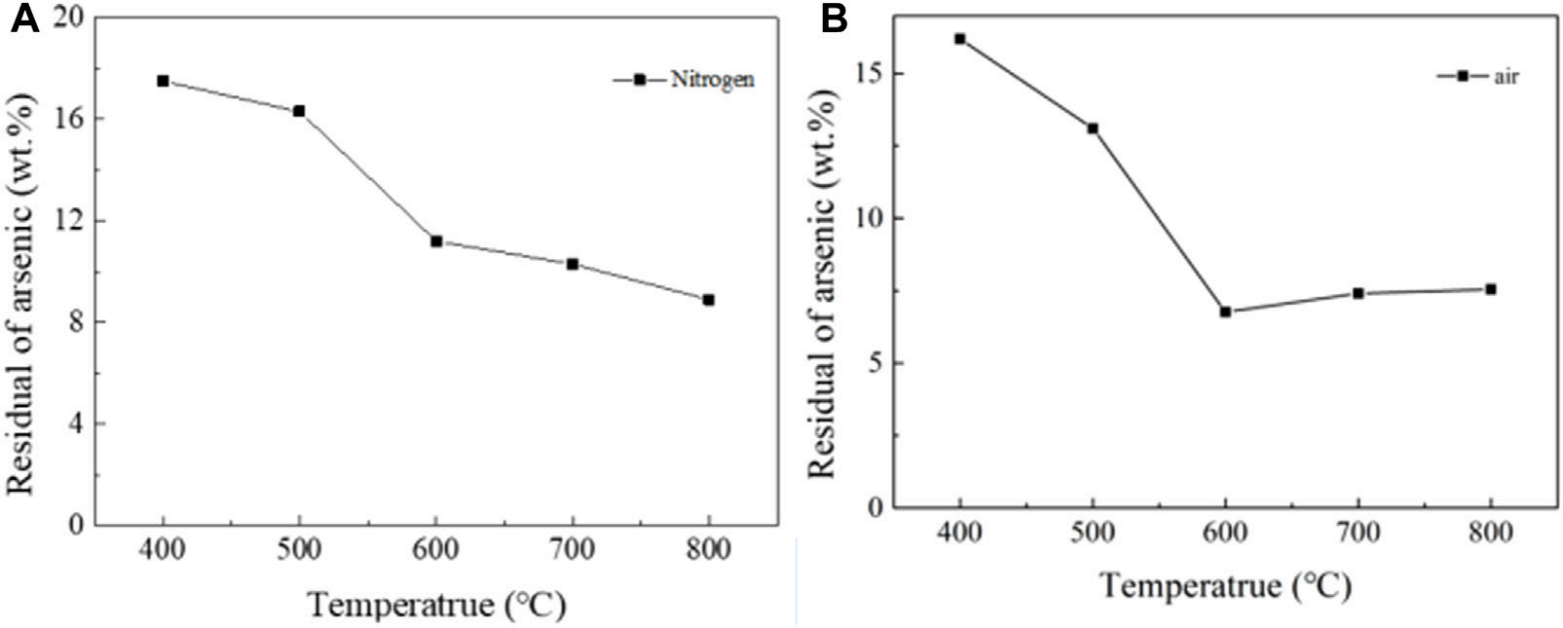
Roasting experiment of NiAs in nitrogen and air. **(A)**, Roasting experiment of NiAs in nitrogen. **(B)**, Roasting experiment of NiAs in air.

The results of NiAs roasting experiment were shown in Figure 6, the roasting conditions were 30 min of time and 1L/min of gas flow rate. When roasting in nitrogen, the arsenic content gradually decreased and reached 8.9 wt% at 800°C, which shows that the effect of roasting without oxygen is poor (Figure 6A). When roasting in air, the arsenic content suddenly decreased when the temperature reached 600°C and kept around 5.5 wt% at subsequent temperatures (Figure 6B). The roasting effect was still not satisfactory. Therefore, direct roasting needs to reduce the roasting of Ni_3_(AsO_4_)_2_ and then oxidize the roasting to remove arsenic. Such a process was repeated to reduce the arsenic content, which makes the direct roasting process for removing arsenic complicated and tedious.

### 3.2 Research on adding sulfide roasting

#### 3.2.1 Reaction mechanism

**FIGURE 7:**
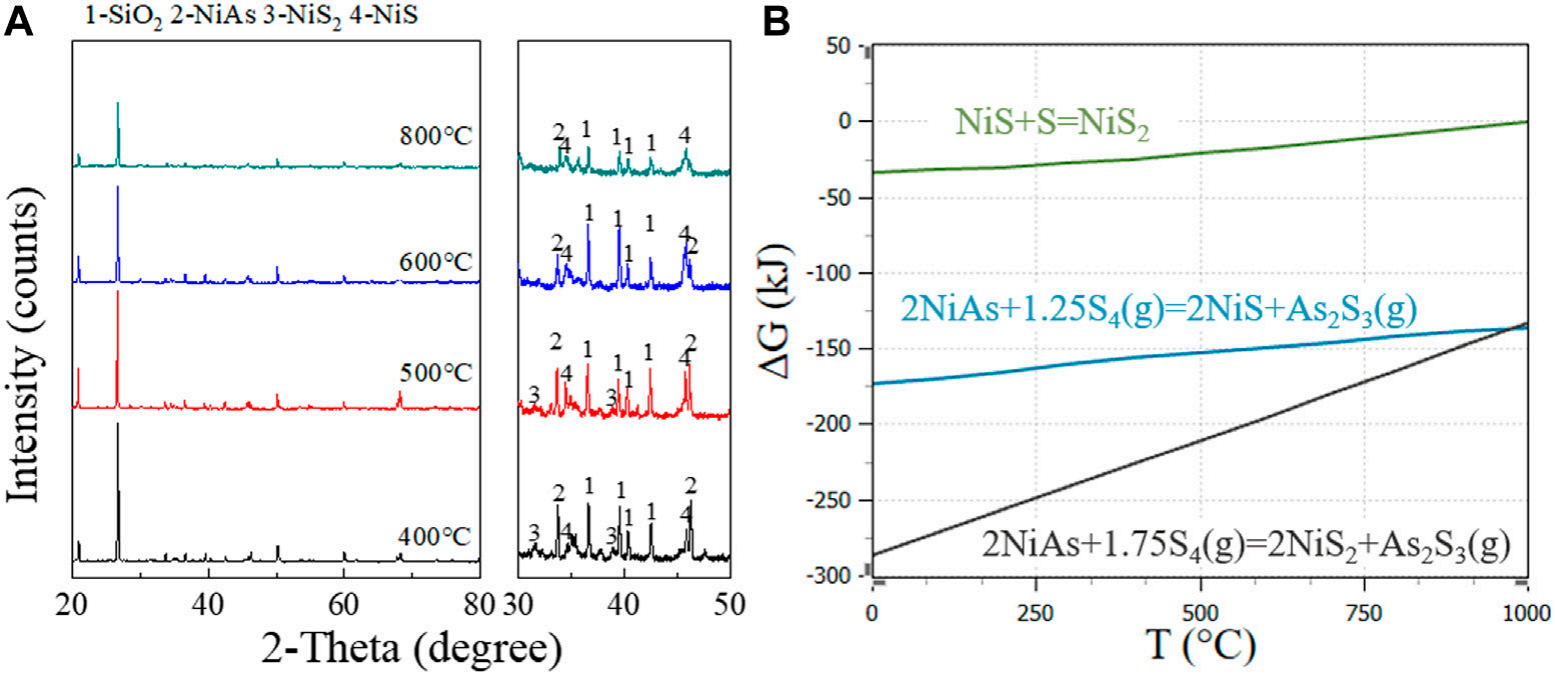
NiS_2_ and NiS formation at different temperatures in sulfidation roasting. **(A)**, XRD patterns for adding sulfide roasting at 800°C, 600°C, 500°C, and 400°C. **(B)**, GFE for adding sulfide reaction of NiAs.

Adding sulfur as a carrier for arsenic volatilization may change the oxidation process to sulfidation process. After sulfidation, the main substances formed are As_2_S_3_(g), NiS, NiS_2_, and S. The roasting process without oxygen prevents the occurrence of arsenic oxidation. Figure 7A shows the substances formed at different temperatures during the sulfidation roasting of the ore. NiS_2_ and NiS are the main products. The reaction equation is inferred as follows (12) and (13).

$$2\text{NiAs}+5/4S_{4}\left(g\right)=2\text{NiS}+{\text{As}}_{2}S_{3}\left(g\right)$$ (12)

$$2\text{NiAs}+7/4S_{4}\left(g\right)=2{\text{NiS}}_{2}+{\text{As}}_{2}S_{3}\left(g\right)$$ (13)

Additionally, sulfur started to break the cycle from S8 when heated to form a long chain, and the chain shortened with increasing temperature and finally boil at 444.6°C. At this time, there were molecules such as S_8_(g), S_6_(g), S_4_(g), S_2_(g) in sulfur vapor. Here, S_4_(g) with the lowest molar GFE was chosen for reaction equation discussion.

From the reaction GFE (Figure 7B), under lower temperature and sufficient sulfur condition, NiS_2_ was preferentially formed. With the increase of temperature, NiS_2_ gradually decreased while NiS increased gradually, which is consistent with the change of peak in the XRD pattern.

#### 3.2.2 Thermodynamic equilibrium component

**FIGURE 8:**
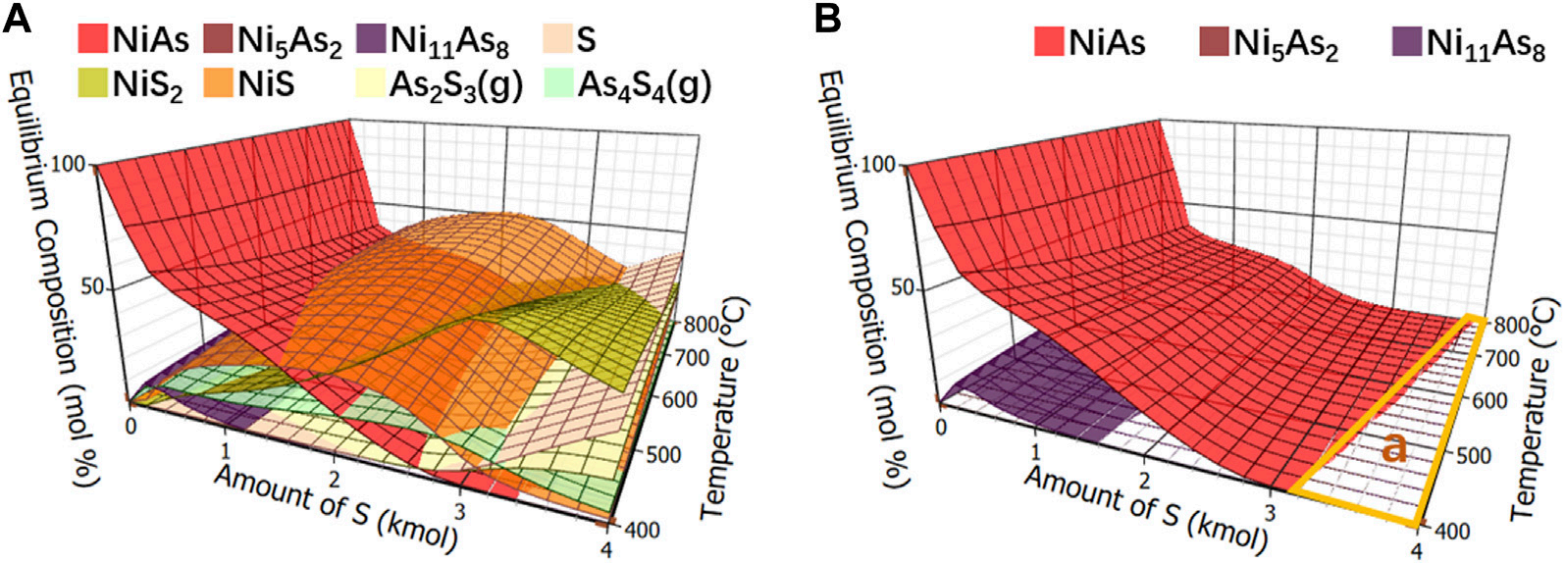
Calculation of equilibrium components in sulfide roasting. **(A)**, Equilibrium component of adding sulfide roasting. **(B)**, Equilibrium component of arsenide nickel and arsenate nickel in adding sulfide roasting.

The calculation results of components in adding sulfide roasting balance were shown in Figure 8A. Other compounds were removed and only arsenide nickel and arsenate nickel were simplified, as shown in Figure 8B. Since there was no oxygen, arsenate nickel was not produced. It was found that when the addition of S is greater than 3 kmol, an area “a” appears where no arsenate nickel or arsenide nickel is produced. Collectively, the arsenic removal of arsenide nickel needs to be carried out under adding sulfide roasting.

#### 3.2.3 Roasting experiment

**FIGURE 9:**
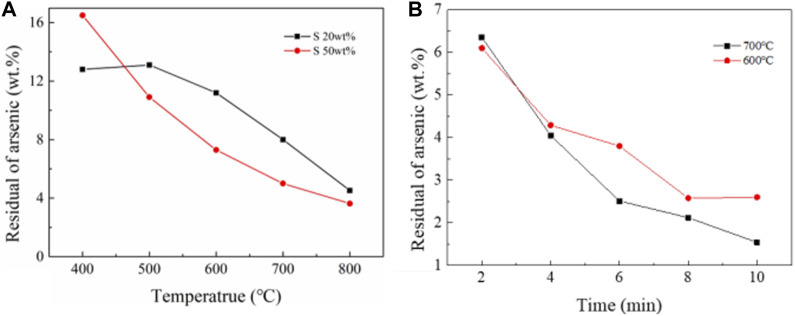
NiAs roasting experiments. **(A)**, Oxidation roasting experiment of NiAs. **(B)**, Reduction roasting experiment of NiAs.

The results of NiAs adding sulfide roasting experiment were shown in Figure 9A. When the roasting conditions were time of 30 min, nitrogen atmosphere, and gas flow rate of 1L/min, the remaining arsenic content decreased gradually with the increase of temperature. And the higher the sulfur content, the better the arsenic removal effect. However, at 400°C, the arsenic removal efficiency of high sulfur content was lower, which was due to the low volatilization rate of sulfur at 400°C and hindered the volatilization of arsenic sulfide. However, when the gas flow rate increased to 2 L/min, the roasting effect with sulfur addition was significantly improved. In Figure 9B, the roasting conditions were nitrogen atmosphere, gas flow rate of 2 L/min, and sulfur content 20 wt%, the reaction was almost completed in 10 min, and the arsenic content dropped to 1.54 wt% at 700°C.

In summary, sulfur is used as reducing agent, and arsenic volatilizes in the form of arsenic sulfide. Due to the poor volatility of arsenic sulfide, high gas flow rate is needed to reduce the partial pressure of arsenic sulfide in the roasting environment. When the gas flow rate is high, roasting with sulfur addition has a good effect on arsenic removal.

### 3.3 Research on adding FeS roasting

#### 3.3.1 Reaction mechanism

**FIGURE 10:**
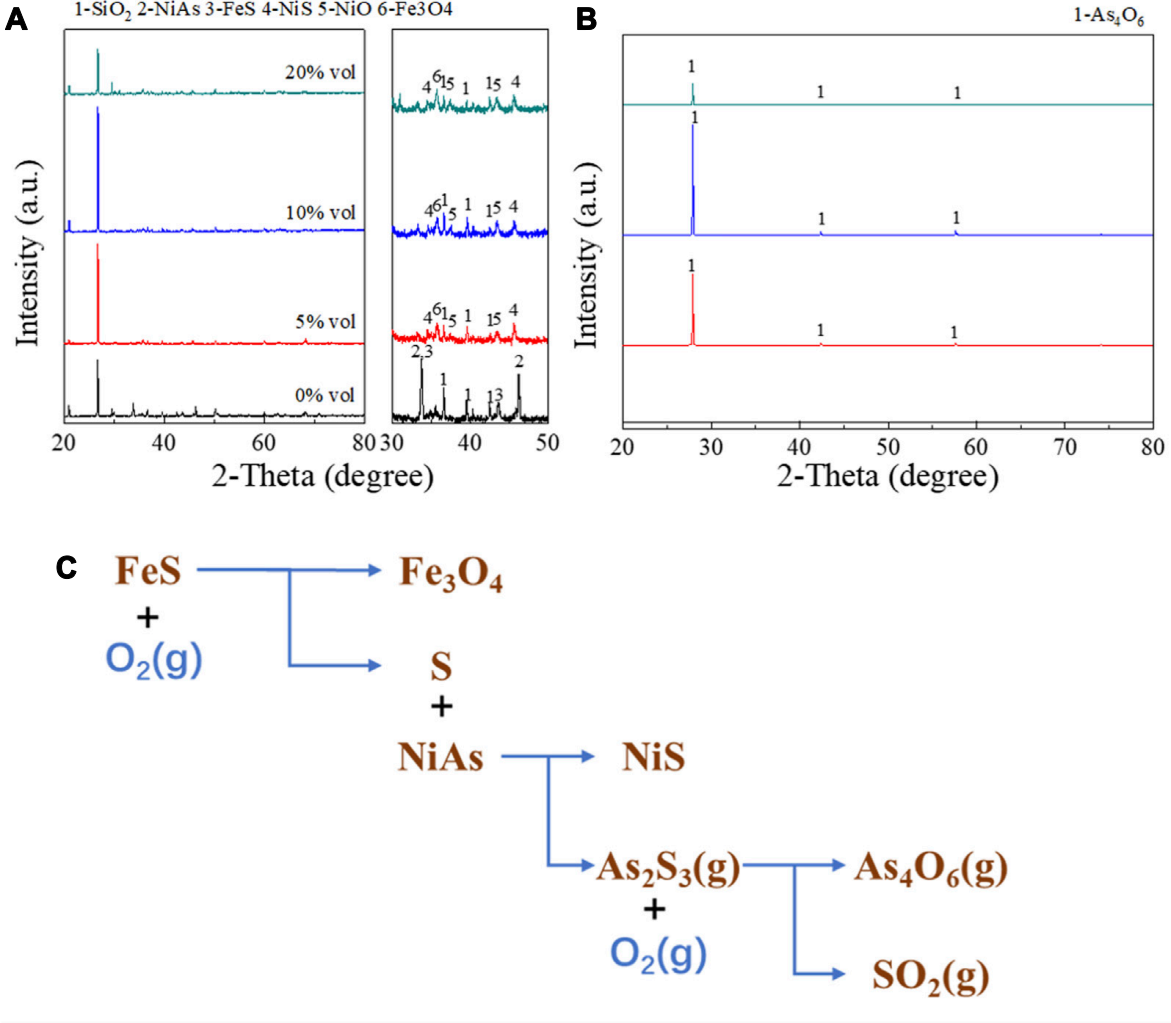
XRD detection on the roasted products and condensates with various oxygen content. **(A)**, XRD pattern of roasted products. **(B)**, XRD pattern of condensates. **(C)**, Reaction mechanism of adding FeS roasting.

In Figure 10, the experiment conducted XRD detection on the roasted products and condensates with oxygen content of 0% vol, 5% vol, 10% vol and 20% vol, with other conditions of 1h roasting time and 1 L/min gas flow rate. The results were shown in Figures 10A, B. When the oxygen content rose to 5%–20%, the roasted products were NiS, NiO, and Fe_3_O_4_, and the condensate was pure As_4_O_6_. This indicated that in this range of oxygen content, arsenic may be removed in the form of As_4_O_6_. And it may be represented by the reaction Eq. 14.

$$4\text{NiAs}+4\text{FeS}+17/3O_{2}\left(g\right)=4\text{NiS}+4/3{\text{Fe}}_{3}O_{4}+{\text{As}}_{4}O_{6}\left(g\right)$$ (14)

However, when there was no oxygen, NiAs and FeS did not react basically, and the condensate obtained was also basically zero. This suggested that FeS cannot react directly with NiAs. Experiments conducted by J.G.Dunn (Dunn, 1997) has explained this issue, where FeS generated S during oxidation and proposed a reaction (15) for this phenomenon.

$$3\text{FeS}+2O_{2}\left(g\right)=3S+2{\text{Fe}}_{3}O_{4}$$ (15)

Therefore, it may be concluded that the reaction (14) is decomposed into reactions (12), reaction (15), and oxidation of arsenic sulfide.

The final process of the roasting with FeS addition was summarized by Figure 10C, and the entire process is divided into three stages. Firstly, the decomposition of FeS occurs, then the sulfuration of NiAs, and finally the oxidation of arsenic sulfide.

#### 3.3.2 Thermodynamic equilibrium component analysis

**FIGURE 11:**
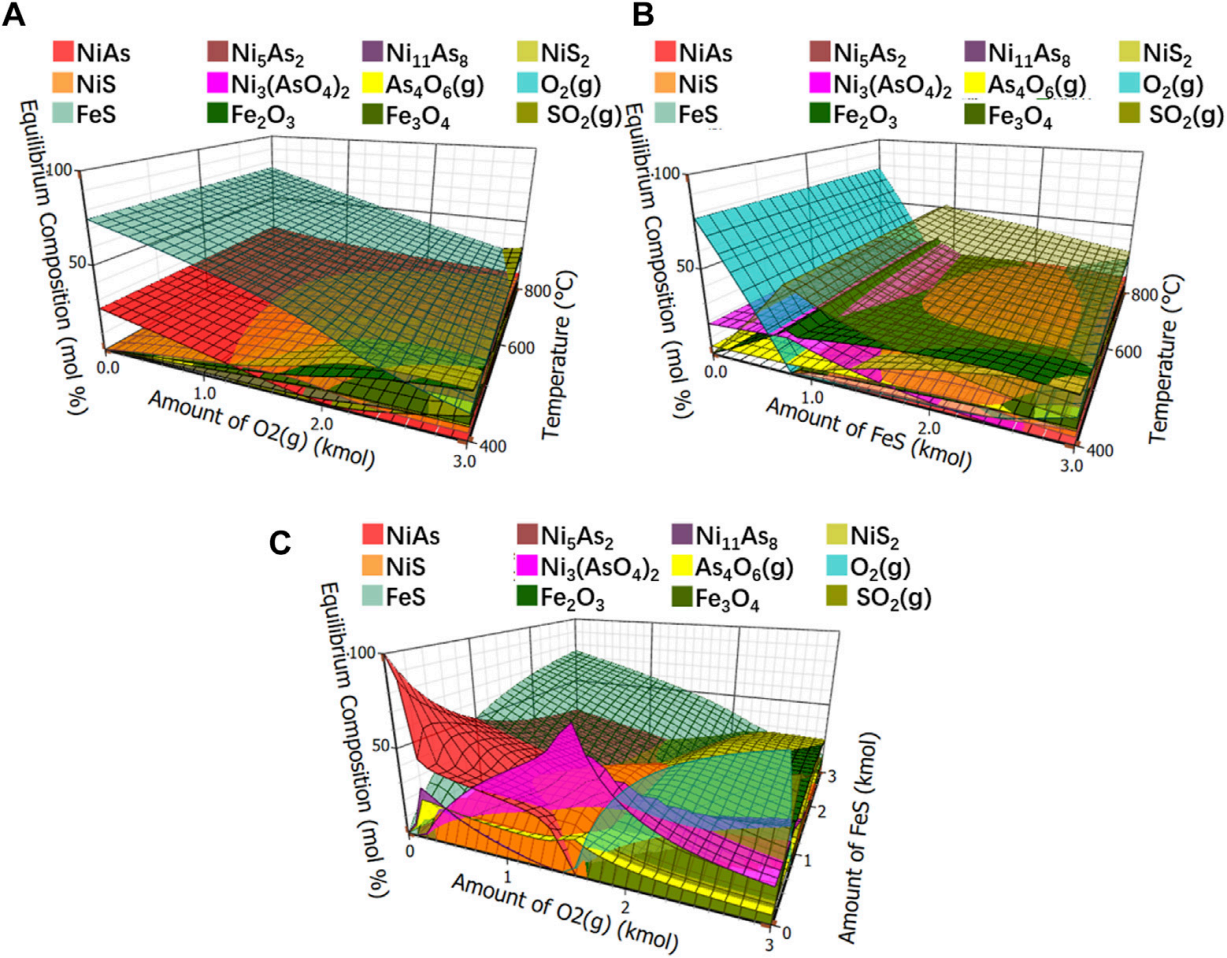
Equilibrium compositions of roasted arsenic nickel ore. **(A)**, Equilibrium component of fixed ratio of NiAs to FeS. **(B)**, Equilibrium component of fixed ratio of NiAs to O_2._ **(C)**, Equilibrium component of fixed temperature to 400°C.

The equilibrium compositions of the roasted arsenic nickel ore after adding FeS were shown in Figure 11. Figure 11A shows the effect of oxygen and temperature on the equilibrium composition with a fixed ratio of NiAs to FeS of 1:3. Figure 11B shows the effect of FeS and temperature on the equilibrium composition with a fixed ratio of NiAs to O_2_ of 1:3. Figure 11C shows the effect of O_2_ and FeS on the equilibrium composition with a fixed temperature of 400°C and a ratio of 1:3.

**FIGURE 12:**
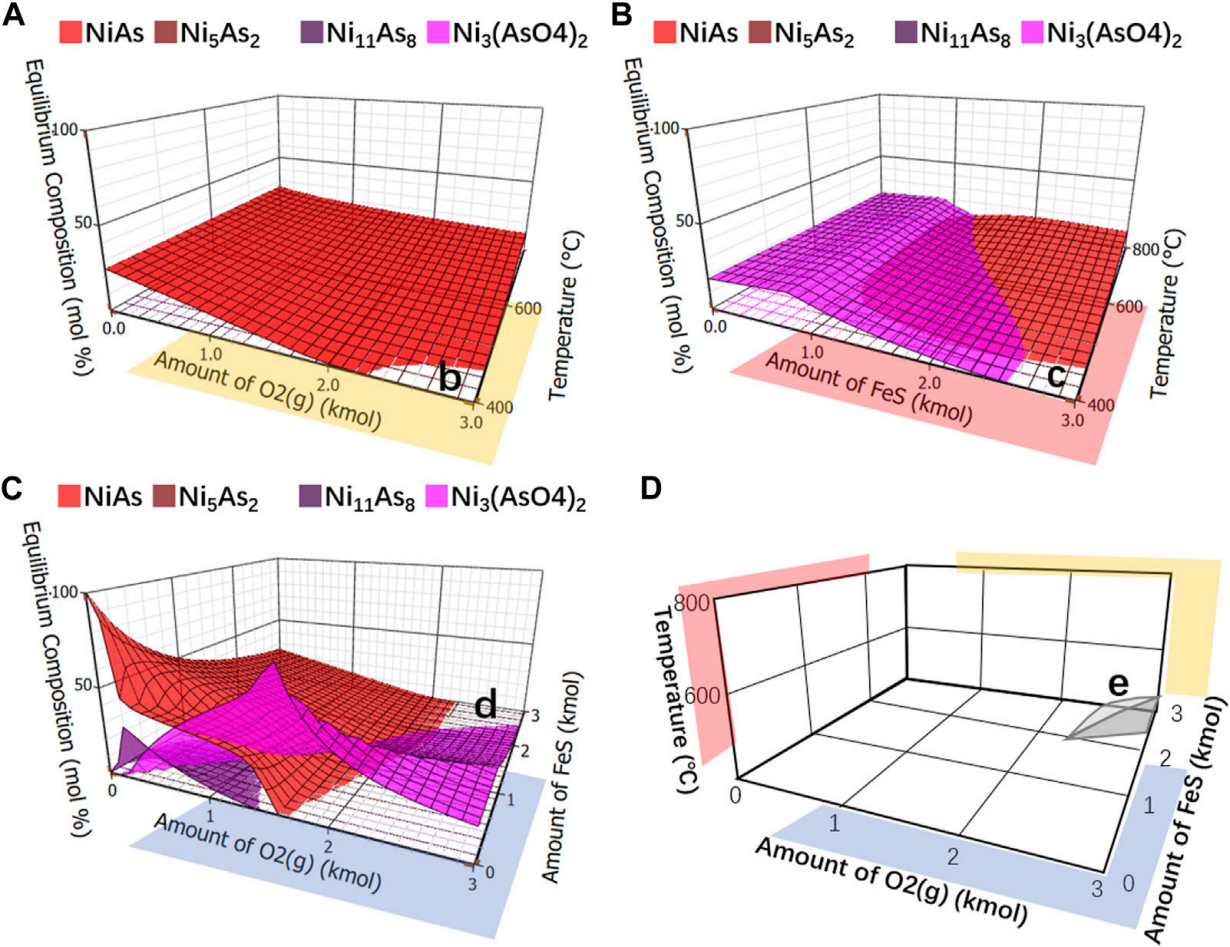
Equilibrium component of arsenide nickel and arsenate nickel. **(A)**, Analysis of arsenide nickel and arsenate nickel O_2_ as x-axis and temperature as y-axis. **(B)**, Analysis of arsenide nickel and arsenate nickel FeS as x-axis and temperature as y-axis. **(C)**, Integrate analysis of arsenide nickel and arsenate nickel O_2_ as x-axis and FeS as y-axis. **(D)**, Merging areas of b, c, **(D)** using temperature as z-axis, FeS as y-axis, and O_2_ as x-axis.

After being removed other compounds and left only arsenic nickel and arsenate nickel, the simplified ones were respectively showed in Figures 12A–C. It was observed blank areas b, c and d in Figures 12A–C, and no arsenate nickel or arsenic nickel was generated in these areas. By merging these areas, with temperature as the z-axis, the amount of FeS as the y-axis and the amount of O_2_ as the x-axis, Figure 12D is formed, in which the three-dimensional area e is the area where no arsenate nickel or arsenic nickel is generated. Observing this area, it may be found that the higher the reaction temperature, the higher the FeS and O_2_ required, which indicates that arsenic needs to be reduced as much as possible to reduce the reaction temperature.

#### 3.3.3 Roasting experiment

**FIGURE 13:**
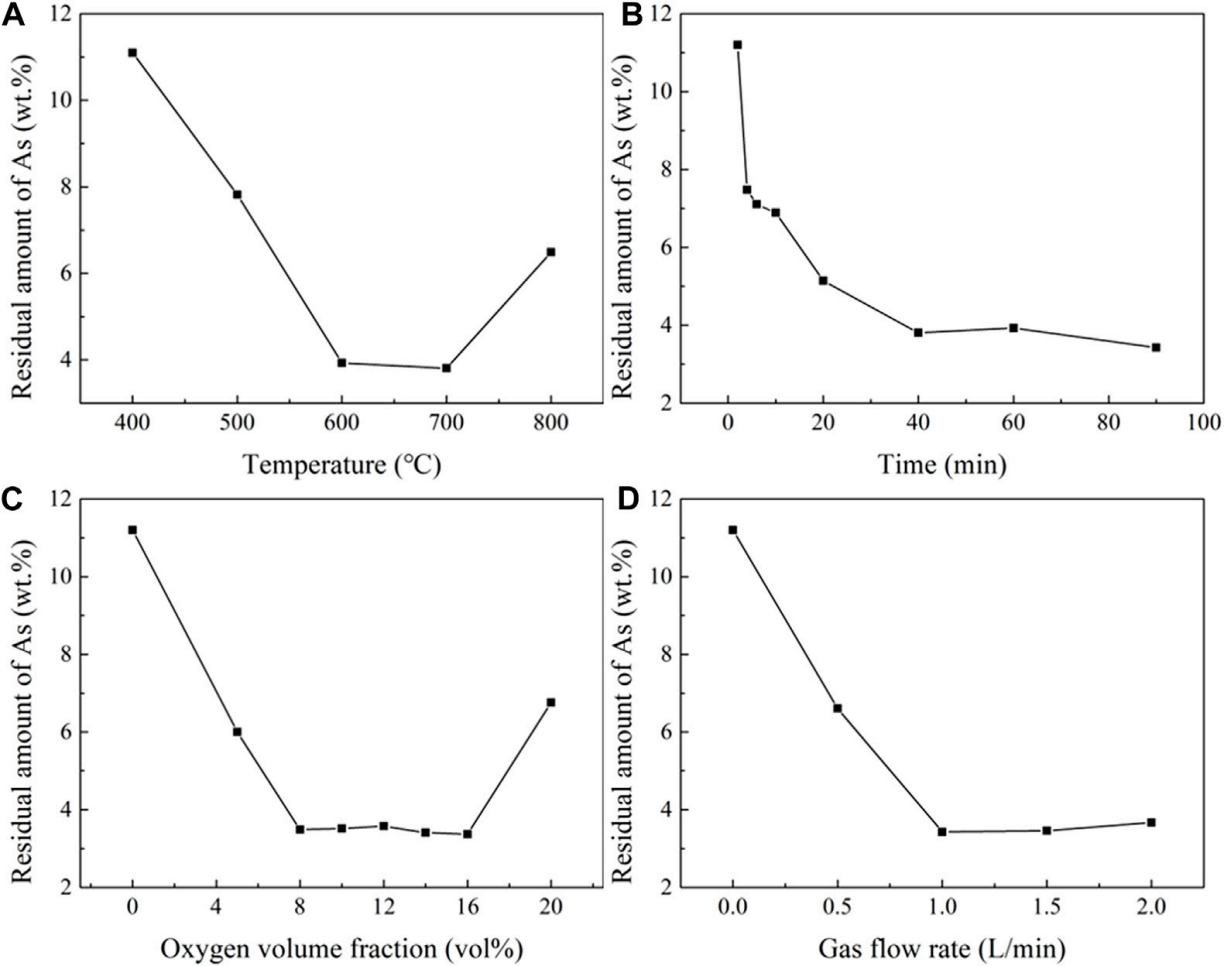
Influence of temperature, time, oxygen, and gas flow velocity on residual arsenic. **(A)**, Effect of temperature (time 60 min, Flow rate 1 L/min, oxygen volume fraction 10 vol%). **(B)**, Effect of time (temperature 600°C, flow rate 1 L/min, oxygen volume fraction 10 vol%). **(C)**, Oxygen volume fraction (temperature 600°C, time 60 min, flow rate 1L/min). **(D)**, Effect of flow rate (temperature 700°C, time 30 min, oxygen volume fraction 10% vol).

The optimizing conditions during roasting experiment were explored with a 5g sample and a ratio of 2:1 between the mineral and FeS. In Figure 13A, the influence of temperature on the residual arsenic content was displayed a rapid decrease as the temperature increased from 500°C to 700°C. However, as the temperature continued to increase, the arsenic content began to rise due to the rapid decrease in the free energy of Ni_11_As_8_. Thus, it is necessary to control the roasting temperature below 600°C, when the residual arsenic content is 3.93 wt%.

In Figure 13B, the influence of time on the residual arsenic content was shown that at a minimum value at 40 min most of the arsenic was removed. When the time was extended to 60 min, the amount of residual arsenic increased due to the continuous volatilization of sulfur in the FeS, and the residual arsenic content dropped to 3.43 wt% when the time reached 90 min.

Figure 13C illustrates the impact of oxygen content on the residual arsenic content. The slight fluctuations were observed in the oxygen content range 8%–16% vol. The lowest residual arsenic content achieved was 3.37 wt% at 16 vol% oxygen content.

Figure 13D shows the effect of gas flow velocity on the residual arsenic content. The lowest residual arsenic content was seen at 3.34 wt% when the gas flow was 1 L/min. However, the residual arsenic content increased when the flow rate exceeded this value, due to the influence of gas flow velocity on the total amount of oxygen. When S was added as the reductant, the residual arsenic content was reduced to 1.54 wt% at 700°C and a gas flow rate of 2.0 L/min. When FeS was added as the reductant, the residual arsenic content was reduced to 3.34 wt% at 600°C, roasting time of 60 min, oxygen volume fraction of 10 vol%, and gas flow rate of 1.0 L/min.

## 4 Conclusion

Through thermodynamic calculation and comparison of oxidation roasting and reduction roasting, it was confirmed that oxidation roasting process cannot be used for arsenic removal from NiAs. Sulfur is employed as a reducing agent, and arsenic volatilizing as arsenic sulfide is produced. As arsenic sulfide has low volatility, a high rate of gas flow is required to reduce the partial pressure of arsenic sulfide in the roasting atmosphere. When the gas flow rate is increased, sulfur addition proves to be effective in removing arsenic.

In the thermodynamic analysis of FeS roasting, the following three points were found: 1) The high roasting temperature produces nickel arsenate or nickel arsenide, which is not conducive to arsenic removal; 2) The addition of oxygen is necessary for FeS reduction roasting process, and the control of oxygen has a great influence on the process; 3) There is an area where nickel arsenate or nickel arsenide should be avoided when FeS and oxygen are in a certain proportion.

Finally, the FeS reduction roasting process is an optimal strategy for for arsenic removal from NiAs. The use of FeS as the reductant significantly reduces the residual arsenic content.

## Data Availability

The original contributions presented in the study are included in the article/Supplementary Material, further inquiries can be directed to the corresponding author.
